# Impact of euploid blastocyst developmental stage and morphological grading on pregnancy outcomes in young recurrent pregnancy loss patients: association with parental chromosomal status

**DOI:** 10.3389/fendo.2025.1644773

**Published:** 2025-09-19

**Authors:** Ruixiao Zhang, Tangmiao Luo, Chenchen Cui, Cuilian Zhang

**Affiliations:** ^1^ Reproductive Medicine Center, Henan Provincial People’s Hospital, Zhengzhou, China; ^2^ Reproductive Medicine Center, People’s Hospital of Zhengzhou University, Zhengzhou, China

**Keywords:** recurrent pregnancy loss, developmental stage, morphological grading, euploid blastocyst transfer, pregnancy outcome, preimplantation genetic testing

## Abstract

**Objective:**

This study investigated the impact of blastocyst developmental stage and morphological grading on pregnancy outcomes following single euploid frozen-thawed blastocyst transfer (SE-FBT) in young patients with recurrent pregnancy loss (RPL) and balanced chromosomal translocations (BCTs), compared to patients with normal karyotypes.

**Methods:**

A retrospective cohort analysis was performed on 449 SE-FBT cycles (2017–2023), comprising 177 cycles from patients with BCT and 272 cycles from patients with normal karyotypes. Blastocysts were categorized according to their developmental stage (day 5 [D5] versus day 6 [D6]) and morphological grading (good versus poor). Multivariable logistic regression models were used to adjust for potential confounders.

**Results:**

Among BCT carriers, D5 blastocysts exhibited significantly higher clinical pregnancy rates (CPR: 83.33% vs. 62.86%; adjusted odds ratio [aOR] = 2.90, *P* = 0.005) and live birth rates (LBR: 75.00% vs. 51.43%; aOR = 2.6, *P* = 0.010) compared to D6 blastocysts, whereas morphological grading showed no significant association after adjustment. Among normokaryotypic patients, however, blastocyst morphological grading was the primary prognostic factor, with good-grade blastocysts yielding superior CPR (74.13% vs. 54.26%, aOR = 2.46, *P* = 0.001) and LBR (56.64% vs. 40.31%, aOR = 1.76, *P* = 0.039), while developmental stage had no significant effect.

**Conclusions:**

These findings suggest that the developmental stage of the blastocyst is the primary determinant of successful outcomes in BCT-associated RPL, whereas embryo morphological grading predominantly influences pregnancy outcomes in RPL patients with normal karyotypes. These results highlight the importance of personalized embryo selection strategies based on parental chromosomal status and embryological characteristics to optimize reproductive outcomes in distinct RPL.

## Introduction

Recurrent pregnancy loss (RPL), defined as two or more consecutive pregnancy losses before 24 weeks of gestation, is a complex clinical challenge with multifactorial etiology (1). Embryonic chromosomal abnormalities constitute a major contributing factor, accounting for 50-60% of cases (2, 3). Advances in assisted reproductive technologies have led to the widespread adoption of preimplantation genetic testing (PGT) for selecting euploid blastocysts, thereby improving pregnancy success rates and reducing miscarriage risk (4, 5). However, even after euploid blastocyst transfer, certain patients—particularly those with RPL—remain susceptible to implantation failure or recurrent miscarriage (6, 7).

Emerging evidence has systematically investigated the effect of euploid blastocyst morphological grading and developmental stage (Day 5 vs. Day 6), on the pregnancy outcomes, as reviewed by Cimadomo et al. (8) Nevertheless, the existing literature presents conflicting conclusions. Some studies indicated a positive correlation between higher morphological grading and live birth rates(LBR) (9–11), while others reported a limited effect of morphological grading on LBR (12–14). Some studies concluded that euploid blastocysts on D5 had a higher live birth rate than those on D6 (15–17), while others showed comparable reproductive competence between the two groups (12, 13) (14, 18). Notably, while existing evidence is predominantly derived from studies involving general infertility populations (11) or well-defined cohorts meeting PGT for aneuploidy/structural rearrangements/monogenic disorders (PGT-A/SR/M) indications, limited data specifically address RPL patients - a clinically distinct population with unique pathophysiological profiles requiring targeted investigation.

In RPL patients, chromosomal abnormalities are detected in 1-5% of cases, among which balanced chromosomal translocations (BCT) represent the predominant cytogenetic anomaly (19). BCT carriers exhibit markedly reduced euploidy rates (26%) (20) compared to the 40% euploid proportion observed in cytogenetically normal counterparts (16). Notably, no studies to date have investigated whether the association between euploid blastocyst characteristics (morphological grading and developmental stage) and transfer outcomes is influenced by the chromosomal status of RPL patients.

The age-associated decline in reproductive capacity is multifactorial, encompassing not only elevated embryonic aneuploidy rates but also metabolic dysregulation and epigenetic reprogramming aberrations (21). While the exact mechanistic relationship between advanced maternal age and endometrial receptivity compromise continues to elicit scientific debate (22). Our investigation specifically focused on young RPL patients (< 38 years) to evaluate the interrelationship between euploid blastocyst parameters, parental chromosomal status, and pregnancy outcomes. This study aimed to establish an evidence-based approach for individualized euploid blastocyst selection in young RPL patients.

## Materials and methods

### Study design and population

This retrospective cohort study was conducted at the Reproductive Medicine Center of Henan Provincial People’s Hospital between January 2017 and September 2023. The study was approved by the Ethics Committee of our hospital. We systematically analyzed clinical data from our hospital’s electronic medical record system for patients with RPL undergoing PGT-A or SR and single euploid frozen-thawed blastocyst transfer (SE-FBT). All enrolled RPL patients with normal parental karyotypes had experienced at least one miscarriage attributable to confirmed embryonic chromosomal abnormalities.

**Figure 1:**
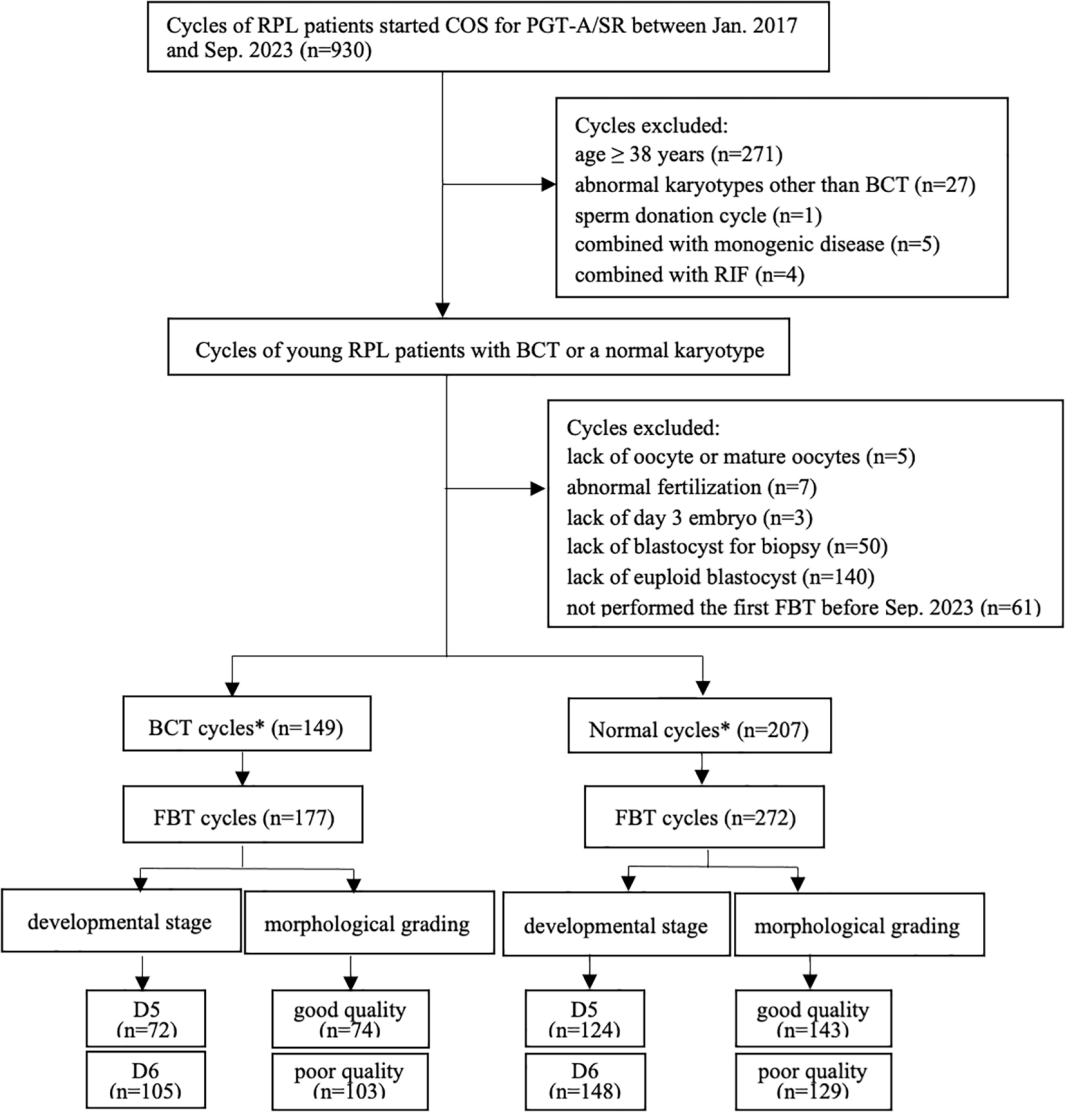
Flowchart of the cycles’ selection. RPL, recurrent pregnancy loss; COS, controlled ovarian stimulation; PGT-A/SR, preimplantation genetic testing for aneuploidy/structural rearrangement; BCT, balanced chromosomal translocations; RIF, repeated implantation failure; FBT, frozen blastocyst transfer. *A PGT cycle has 1 FBT cycles.

The inclusion criteria comprised: 1) Couples where either partner carried BCT, including reciprocal and Robertsonian translocations or both had normal karyotypes; 2) Female partners aged < 38 years at cycle initiation; 3) Completion of at least one SE-FBT cycle involving day5/6 blastocysts. Exclusion criteria included: 1) Advanced maternal age (AMA ≥ 38 years); 2) Non-BCT chromosomal abnormalities in either partner; 3) Use of donor gametes in PGT-A/SR cycles; 4) Concomitant monogenic disorders or recurrent implantation failure (RIF); 5) Cases without euploid blastocysts available or incomplete first FBT before September 2023. The patient selection algorithm is detailed in **Figure 1**. Notably, mosaic blastocysts were systematically excluded from transfer protocols.

Prior to treatment initiation, all participants received comprehensive genetic counseling regarding PGT’s clinical implications, technical limitations, and potential risks. Written informed consent for PGT procedures was obtained from all couples following institutional ethical guidelines.

### Treatment protocols

#### Ovarian stimulation and embryo culture

Three established controlled ovarian stimulation (COS) protocols were employed: 1) GnRH agonist long protocol (early follicular phase long-acting or mid-luteal phase short-acting); 2) Flexible GnRH antagonist protocol; 3) Progestin-primed ovarian stimulation (PPOS) protocol. Specific protocol parameters followed published methodologies (23, 24). Individualized COS regimens were formulated based on comprehensive clinical parameters: maternal age, body mass index (BMI), antral follicle count (AFC), anti-Müllerian hormone (AMH) levels, baseline follicular status, prior ovarian response, and socioeconomic considerations. Gonadotropin dosing was dynamically adjusted through serial transvaginal ultrasonography (TVUS) and serum estradiol (E_2_) monitoring. Ovulation triggering criteria required ≥ 1 follicle ≥ 18mm or ≥ 2 follicles ≥ 17mm, with human chorionic gonadotropin (hCG) (4000-10000IU, Lizhu Pharmaceutical Trading) and/or GnRH agonist (0.1/0.2mg, triptorelin acetate, Ferring) based on follicular cohort characteristics and peak E2 levels. Oocyte retrieval was performed 34–37 hours post-trigger under TVUS guidance.

#### Embryo laboratory procedures

For all cycles, intracytoplasmic sperm injection (ICSI) was performed 4–6 h post-oocyte retrieval. Fertilization confirmation (two pronuclei) occurred 16–18 h post-ICSI. Embryos were cultured to blastocyst stage using Vitrolife sequential medium (Goteborg, Sweden) under controlled conditions (37°C, 6% CO_2_, 5% O_2_, 89% N_2_), with blastocyst grading preceding biopsy. Blastocyst morphology was independently assessed by two experienced embryologists using Gardner’s criteria (25), evaluating expansion degree (stage 1-6), inner cell mass (ICM), and trophectoderm (TE) morphology (for stage 3-6). Expansion stages were: 1 (Early; blastocoel < 50% embryo volume), 2 (blastocoel ≥ 50%), 3 (Full; blastocoel fills embryo completely), 4 (Expanded; blastocoel > early embryo volume, thinning zona), 5 (Hatching; trophectoderm herniating through zona), and 6 (Hatched; blastocyst fully escaped from zona). ICM was scored: A (dense, numerous cells), B (loose grouping, moderate cells), or C (sparse cells). TE was scored: A (cohesive, multicellular epithelium), B (fragmented epithelium, few cells), or C (minimal, enlarged cells). Embryos were categorized as: good quality: ≥ 3BB grade; poor quality: 3–6 AC/BC/CA/CB grades (13, 26).

TE biopsy was conducted on blastocysts meeting the minimum quality threshold (≥ 3BC/CB) using a laser-assisted system. For each blastocyst, 5–10 cells were biopsied. Following biopsy, all blastocysts were cryopreserved via vitrification. Genetic analysis for aneuploidy was performed using next-generation sequencing (NGS) on the MiSeqDx platform (Illumina, San Diego, CA, USA). Whole-genome amplification of biopsied cells was carried out with the SurePlex kit, followed by library preparation using the VeriSeq PGS Kit (Illumina), in strict accordance with the manufacturer’s instructions. In PGT-SR cases, next-generation sequencing with microdissecting junction region (MicroSeq) analysis was subsequently employed to differentiate non-carrier from balanced carrier embryos, as previously described (27).

### Single euploid blastocyst transfer protocols

Prior to SE-FBT, all patients underwent standardized evaluations and treatments for RPL in accordance with established clinical guidelines (1). Patients with confirmed euploid blastocysts underwent SE-FBT, following three standardized endometrial preparation protocols: 1) Natural cycle (NC): Indicated for ovulatory women with regular cycles. Endometrial development was monitored by TVUS from cycle day 10. Progesterone was administered on the day of ovulation, followed by embryo transfer 5 days later; 2) Hormone replacement therapy (HRT): Designed for anovulatory women or those requiring controlled endometrial preparation. Oral estradiol valerate tablets were given at 2–4 mg daily starting on cycle day 2-4, with subsequent dose titration (4–8 mg daily) based on serial TVUS assessments for a minimum of 11 days. Endometrial thickness ≥ 8 mm or maximum estrogen exposure duration of 21 days triggered progesterone initiation, followed by transfer 6 days thereafter.; 3) GnRHa-HRT: Reserved for patients with implantation failure or endometriosis. Pituitary downregulation was achieved with single-dose GnRHa (Decapeptyl^®^ 3.75 mg, Ferring) administered on cycle day 2-4. Following 14 days of downregulation (confirmed by serum estradiol < 50 pg/mL and absence of ovarian cysts > 10 mm), endometrial preparation proceeded as HRT protocol. Luteal phase support comprised oral dydrogesterone (Duphaston^®^ 10mg twice daily, Abbott) and vaginal progesterone gel (Crinone^®^ 90mg once daily, Merck Serono), maintained until 10 gestational weeks.

### Pregnancy outcome assessment

Serum β-hCG levels were measured 14 days after ET. β-hCG ≥ 25 IU/L was considered indicative of a positive result. TVUS was performed 4–5 weeks after transfer. Outcome measures were calculated as follows: 1) Clinical pregnancy rate (CPR): number of clinical pregnancies/total number of transfer cycles × 100%; 2) Early miscarriage rate (EMR): number of the clinical pregnancy losses <12 gestational weeks/total number of clinical pregnancies × 100%; 3) Live birth rate (LBR): number of deliveries with ≥ 1 liveborn infant at ≥ 24 weeks’ gestation/total embryo transfer cycles) × 100%. Multifetal deliveries occurring within a single gestational cycle were documented as a single live birth event.

### Statistical analysis

Statistical analyses were conducted using IBM SPSS Statistics (version 27.0; IBM Corp). Continuous variables were initially evaluated for normality distribution through the Shapiro-Wilk test, and were expressed as mean ± standard deviation (SD) or median (quartile 1-quartile 3) [M (P_25_, P_75_)]. Parametric and non-parametric tests, including Student’s t-test and Mann-Whitney U test, were used for comparative analyses. Categorical variables were presented as frequency counts with percentages (%) and analyzed using the Pearson’s Chi-square test. To account for the fact that multiple data points were derived from the same patient, data analysis was performed using generalized estimating equations (GEE). Multivariable logistic regression analysis was performed to assess the effect of embryo developmental stage and morphological grading on the pregnancy outcomes. Potential confounding variables were selected on the basis of univariable regression analyses with a significance level of *P* < 0.2 and a review of the existing literature. Results were expressed as adjusted odds ratio (aOR) and 95% confidence intervals (95%CI). *P* < 0.05 was considered statistically significant.

## Results

### Study population

As shown in **Figure 1**, a total of 177 FBT cycles after 149 PGT cycles of RPL patients with BCT (BCT cycles) and a total of 272 FBT cycles derived from 207 PGT cycles of RPL patients with normal karyotypes (normal cycles) were finally included in this study. We analyzed CPR, EMR and LBR based on developmental stage (D5 and D6) and morphological grading (good quality and poor quality). In the BCT cycles, 28 patients underwent two FBT cycles, while in the normal cycles, 38 patients had two FBT cycles, 12 patients had three FBT cycles, and 1 patient underwent four FBT cycles.

### BCT cycles

**Table 1** presents the baseline characteristics of the PGT cycles in RPL patients with BCT. The subtypes of BCT for all included patients are detailed in **Supplementary Table 1**. **Table 2** summarizes the parameters of FBT cycles. Comparative analysis revealed no statistically significant differences in maternal age, BMI, number of previous miscarriages, endometrial thickness, or endometrial preparation protocol when comparing the D5 vs. D6 transfer groups (*P >* 0.05) and good vs. poor quality groups (*P* > 0.05). Notably, morphological grading demonstrated a significant disparity between D5 and D6 blastocysts (*P* = 0.001), with the D5 group exhibiting a higher proportion of good quality blastocysts (55.56% vs. 32.38%). Meanwhile, developmental stage analysis showed a significant difference between good and poor quality blastocysts (*P* = 0.001), where good quality blastocysts were predominantly observed in the D5 group (54.05% vs. 31.07%).

**Table 1:** Baseline characteristics of the PGT cycles in RPL patients with balanced chromosomal translocations.

Parameters	Value
Cycles (n)	149
Maternal age (years)	29 (26,31)
Paternal age (years)	29 (27,32)
Maternal BMI (kg/m^2^)	23.23 (21.1,25.97)
Paternal BMI (kg/m^2^)	25.46 ± 3.62
AFC (n)	15 (12,20)
Basal FSH (mIU/ml)	6.39 (5.47,7.27)
Basal E_2_ (pg/ml)	35.17 (28.67,43.96)
AMH (ng/ml)	3.53 (2.52,5.09)
No. of prior pregnancies (n)	
2	82 (55.03)
3	39 (26.17)
4	16 (10.74)
5	6 (4.03)
≥6	6 (4.03)
No. of previous miscarriages (n)	
2	97 (65.1)
3	36 (24.16)
4	11 (7.38)
≥5	5 (3.36)
PCOS	
Yes	10 (6.71)
No	139 (93.29)
Type of COS protocols	
Antagonist	38 (25.5)
PPOS	29 (19.46)
Long	82 (55.03)
Total dosage of Gn used (IU)	2100 (1650,2625)
Duration of Gn used (day)	10 (9,11)
E_2_ level on trigger day (pg/ml) ^a^	1810 (1171,2615)
Endometrial thickness on trigger day (mm)	9 (7,10)
No. of oocytes retrieved per cycle (n)	14 (10,18)
MII rate per cycle (%)	85.71 (73.33,95.24)
2PN rate per cycle (%)	83.33 (75,93.75)
Cleavages rate per cycle (%)	90.91 (83.33,100)
Blastocyst formation rate per cycle (%)	60 (45.45,75)
No. of blastocyst biopsied per cycle (n)	5 (3,7)
No. of euploid embryos per cycle (n)	2 (1,2)

BMI, body mass index; AFC, antral follicle count; FSH, follicle stimulating hormone; E_2_, estradiol; AMH, anti-Mullerian hormone; PCOS, polycystic ovary syndrome; COS, controlled ovarian stimulation; PPOS, progestin-primed ovarian stimulation; Gn, gonadotropin; PN, pronucleus.

^a^A total of 19 cycles with E_2_ levels above 3000 pg/ml and no specific values were recorded and therefore excluded from the statistical description.

**Table 2:** Characteristics of frozen-thawed embryo transfer cycles in PRL patients with balanced chromosomal translocations.

Parameters	Developmental stage		*P* value	Morphological grading		*P* value
	D5 (n=72)	D6 (n=105)		Good quality (n=74)	Poor quality (n=103)	
Maternal age (years)	28 (26, 31.5)	30 (27, 32)	0.230	29.5 (27, 31)	30 (27, 32)	0.688
Maternal BMI (kg/m^2^)	23.59 (21.51, 26.11)	23.15 (21.1, 26.04)	0.641	23.38 (21.8, 26.04)	23.4 (21.08, 26.17)	0.646
No. of previous miscarriages (n)						
2	50 (69.44)	65 (61.9)	ref	45 (60.81)	70 (67.96)	ref
3	15 (20.83)	28 (26.67)	0.405	21 (28.38)	22 (21.36)	0.261
4	5 (6.94)	7 (6.67)	0.966	7 (9.46)	5 (4.85)	0.136
≥5	2 (2.78)	5 (4.76)	0.461	1 (1.35)	6 (5.83)	0.116
Endometrial thickness (mm)	9 (8, 9.9)	8.9 (8.1, 9.9)	0.975	9 (8, 10)	9 (8.2, 9.7)	0.240
Endometrial preparation						
GnRHa-HRT	13 (18.06)	27 (25.71)	0.348	20 (27.03)	20 (19.42)	0.348
HRT	58 (80.56)	73 (69.52)	0.210	54 (72.97)	77 (74.76)	0.210
NC	1 (1.39)	5 (4.76)	ref	0 (0)	6 (5.83)	ref
Developmental stage						0.001*
D5				40 (54.05)	32 (31.07)	
D6				34 (45.95)	71 (68.93)	
Morphological grading			0.001*			
Good quality	40 (55.56)	34 (32.38)				
Poor quality	32 (44.44)	71 (67.62)				

GnRHa, gonadotropin-releasing hormone agonist; HRT, hormone replacement treatment; NC, natural cycle.

**Table 3** delineates the associations among blastocyst developmental stages, morphological grading, and pregnancy outcomes. D5 blastocysts demonstrated significantly superior CPR (83.33% vs. 62.86%, *P* = 0.003) and LBR (75.00% vs. 51.43%, *P* = 0.003) compared with D6 blastocysts. These associations retained statistical significance following multivariable adjustment (aOR = 2.90, 95% CI = 1.37-6.13, *P* = 0.005 for CPR; aOR = 2.60, 95% CI = 1.26-5.35, *P* = 0.010 for LBR). EMR showed no intergroup disparity (8.33% vs. 12.12%, *P* = 0.502).

**Table 3:** The association between pregnancy outcomes, blastocyst developmental stage and morphological grading in RPL patients with balanced chromosomal translocations.

	Outcome	OR (95% CI)	*P* value	aOR (95% CI)	*P* value
CPR, n (%)					
Developmental stage					
D5	60/72 (83.33)	2.97 (1.44-6.11)	0.003*	2.90 (1.37-6.13)	0.005*
D6	66/105 (62.86)	ref		ref	
Morphological grading					
Good quality	60/74 (81.08)	2.42 (1.20-4.85)	0.013*	1.80 (0.84-3.84)	0.129
Poor quality	66/103 (64.08)	ref		ref	
EMR, n (%)					
Developmental stage					
D5	5/60 (8.33)	0.67 (0.21-2.15)	0.502	—	
D6	8/66 (12.12)	ref		ref	
Morphology					
Good quality	5/60 (8.33)	0.67 (0.21-2.12)	0.493	—	
Poor quality	8/66 (12.12)	ref		ref	
LBR, n (%) ^a^					
Developmental stage					
D5	54/72 (75.00)	2.83 (1.44-5.56)	0.003*	2.6 (1.26-5.35)	0.010*
D6	54/105 (51.43)	ref		ref	
Morphological grading					
Good quality	53/74 (71.62)	2.2 (1.19-4.09)	0.012*	1.66 (0.88-3.14)	0.116
Poor quality	55/103 (53.40)	ref		ref	

CPR, clinical pregnancy rate; EMR, early miscarriage rate; LBR, live birth rate; OR, odds ratio; CI, confidence interval; aOR, adjusted odds ratio.

Adjust for: maternal age, BMI, AMH levels, number of previous miscarriages, endometrial preparation, endometrial thickness, developmental stage/morphological grading.

^a^Four developed into monozygotic twins. *: The difference is statistically significant.

*The difference is statistically significant.

Notably, while unadjusted analysis revealed significant outcome differences between blastocyst morphological grading groups (CPR: 81.08% vs. 64.08%, *P* = 0.013; LBR: 71.62% vs. 53.40%, *P* = 0.012 for good vs. poor quality, respectively), these associations were attenuated after controlling for confounders (CPR: aOR = 1.80, 95% CI = 0.84-3.84, *P* = 0.129; LBR: aOR = 1.66, 95% CI = 0.88-3.14, *P* = 0.116). EMR remained comparable across quality categories (8.33% vs. 12.12%, *P* = 0.493). Adjustment models incorporated maternal age, BMI, AMH levels, number of previous miscarriages, endometrial preparation protocols, endometrial thickness, and blastocyst developmental stage/morphological grading.

We further analyzed the combined impact of developmental stage and morphological grading on pregnancy outcomes (**Supplementary Table 2**). Our analysis revealed that developmental day significantly impacts pregnancy outcomes specifically among poor-quality blastocysts.

### Normal cycles

**Table 4** presents the baseline characteristics of the PGT cycles in normokaryotypic RPL patients, while **Table 5** details the parameters of FBT cycles. No significant intergroup differences were observed in maternal age, BMI, number of previous miscarriages, endometrial thickness or endometrial protocol when stratified by developmental stage and morphological grading (all *P* > 0.05).

**Table 4:** Baseline characteristics of the PGT cycles in normokaryotypic RPL patients.

Parameters	Value
Cycles (n)	207
Maternal age (years)	32 (30,34)
Paternal age (years)	32 (30,35)
Maternal BMI (kg/m^2^)	22.83 (20.7,24.6)
Paternal BMI (kg/m^2^)	25.65 (23.2,28.09)
AFC (n)	14 (10,19)
Basal FSH (mIU/ml)	6.29 (5.45,7.1)
Basal E_2_ (pg/ml)	39.18 (30.97,49.28)
AMH (ng/ml)	3.25 (2.02,4.72)
No. of prior pregnancies (n)	
2	40 (19.32)
3	76 (36.71)
4	40 (19.32)
5	23 (11.11)
≥6	28 (13.53)
No. of previous miscarriages (n)	
2	70 (33.82)
3	83 (40.1)
4	36 (17.39)
≥5	18 (8.7)
PCOS	
Yes	24 (11.59)
No	183 (88.41)
Type of COS protocols	
Antagonist	69 (33.33)
PPOS	51 (24.64)
Long	87 (42.03)
Total dosage of Gn used (IU)	2100 (1725,2525)
Duration of Gn used (day)	9 (8,11)
E_2_ level on trigger day (pg/ml) ^a^	1700 (1112,2450)
Endometrial thickness on trigger day (mm)	9 (7,10)
No. of oocytes retrieved per cycle (n)	12 (7,17)
MII rate per cycle (%)	83.33 (71.43,96.55)
2PN rate per cycle (%)	83.33 (71.43,100)
Cleavages rate per cycle (%)	94.44 (80,100)
Blastocyst formation rate per cycle (%)	64.29 (50,80)
No. of blastocyst biopsied per cycle (n)	4 (3,6)
No. of euploid embryos per cycle (n)	2 (1,3)

BMI, body mass index; AFC, antral follicle count; FSH, follicle stimulating hormone; E_2_, estradiol; AMH, anti-Mullerian hormone; PCOS, polycystic ovary syndrome; COS, controlled ovarian stimulation; PPOS, progestin-primed ovarian stimulation; Gn, gonadotropin; PN, pronucleus.

^a^A total of 24 cycles with E_2_ levels above 3000 pg/ml and no specific values were recorded and therefore excluded from the statistical description.

**Table 5:** Characteristics of frozen-thawed embryo transfer cycles in normokaryotypic RPL patients.

Parameters	Developmental stage		*P* value	Morphological grading		*P* value
	D5 (n=124)	D6 (n=148)		Good quality (n=143)	Poor quality (n=129)	
Maternal age (years)	32.5 (29.5, 35)	32 (30,35)	0.883	32 (30, 35)	32 (29, 35)	0.900
Maternal BMI (kg/m^2^)	22.83 (20.32, 24.64)	23.11 (21.23,25.04)	0.149	22.89 (20.32, 24.98)	23.13 (21.6, 24.69)	0.151
No. of previous miscarriages (n)						
2	43 (34.68)	45 (30.41)	ref	41 (28.67)	47 (36.43)	ref
3	40 (32.26)	69 (46.62)	0.168	63 (44.06)	46 (35.66)	0.246
4	27 (21.77)	25 (16.89)	0.795	28 (19.58)	24 (18.6)	0.661
≥5	14 (11.29)	9 (6.08)	0.423	11 (7.69)	12 (9.3)	0.934
Endometrial thickness (mm)	8.5 (7.5, 10.0)	8.95 (8,10)	0.301	9 (8, 10)	8.5 (7.6, 9.5)	0.424
Endometrial preparation						
GnRHa-HRT	33 (26.61)	37 (25.00)	0.302	36 (25.17)	34 (26.36)	0.217
HRT	85 (68.55)	100 (67.57)	0.280	100 (69.93)	85 (65.89)	0.135
NC	6 (4.84)	11 (7.43)	ref	7 (4.9)	10 (7.75)	ref
Developmental stage						0.024*
D5				74 (51.75)	50 (38.76)	
D6				69 (48.25)	79 (61.24)	
Morphological grading			0.022*			
Good quality	74 (59.68)	69 (46.62)				
Poor quality	50 (40.32)	79 (53.38)				

GnRHa, gonadotropin-releasing hormone agonist; HRT, hormone replacement treatment; NC, natural cycle.

Comparative analysis revealed significant morphological disparities between D5 and D6 blastocysts (*P* = 0.022), with D5 blastocysts demonstrating a higher prevalence of good quality blastocysts (59.68% vs. 46.62%). Importantly, developmental stage distribution differed significantly between blastocysts with good vs. poor quality (*P* = 0.024), where 51.75% of good quality blastocysts were derived from D5 compared to 38.76% in the poor quality group.

**Table 6** presents the association analysis of blastocyst developmental stages and morphological grading with pregnancy outcomes. No significant differences were observed between D5 and D6 blastocysts (68.55% vs. 61.49%, *P* = 0.226; 50.00% vs. 47.97%, *P* = 0.815, respectively) regarding for CPR and LBR. These non-significant associations persisted after multivariable adjustment (aOR = 1.16, 95% CI = 0.70-1.95, *P* = 0.560 for CPR; aOR = 1.01, 95% CI = 0.62-1.65, *P* = 0.968 for LBR). Notably, EMR were comparable between D5 and D6 groups (20.00% vs. 20.88%, *P* = 0.882).

**Table 6:** The association between pregnancy outcomes, blastocyst developmental stage and morphological grading in normokaryotypic RPL patients.

	Outcome	OR (95% CI)	*P* value	aOR (95% CI)	*P* value
CPR, n (%)					
Developmental stage					
D5	85/124 (68.55)	1.36 (0.83-2.26)	0.226	1.16 (0.70-1.95)	0.560
D6	91/148 (61.49)	ref		ref	
Morphological grading					
Good quality	106/143 (74.13)	2.46 (1.47-4.1)	<0.001*	2.46 (1.46-4.15)	<0.001*
Poor quality	70/129 (54.26)	ref		ref	
EMR, n (%)					
Developmental stage					
D5	17/85 (20.00)	0.94 (0.44-2.02)	0.882	—	
D6	19/91 (20.88)	ref		ref	
Morphological grading					
Good quality	20/106 (18.87)	0.88 (0.4-1.93)	0.755	—	
Poor quality	16/70 (22.86)	ref		ref	
LBR, n (%)^a^					
Developmental stage					
D5	62/124 (50.00)	1.06 (0.66-1.7)	0.815	1.01 (0.62-1.65)	0.968
D6	71/148 (47.97)	ref		ref	
Morphological grading					
Good quality	81/143 (56.64)	1.77 (1.06-2.98)	0.031*	1.76 (1.03-3.01)	0.039*
Poor quality	52/129 (40.31)	ref		ref	

CPR, clinical pregnancy rate; EMR, early miscarriage rate; LBR, live birth rate; OR, odds ratio; CI, confidence interval; aOR, adjusted odds ratio.

Adjust for: maternal age, BMI, AMH, number of previous miscarriages, endometrial preparation, endometrial thickness, developmental stage/morphological grading.

^a^One developed into monozygotic twins. *: The difference is statistically significant.

*The difference is statistically significant.

In contrast, blastocyst morphological grading significantly impacted reproductive outcomes. The good-quality blastocyst group exhibited markedly higher CPR and LBR compared with the poor-quality group (74.13% vs. 54.26%, *P* < 0.001; 56.64% vs. 40.31%, *P* = 0.031, respectively). These associations remained statistically significant after adjusting for potential confounders (aOR = 2.46, 95% CI = 1.46-4.15, *P* < 0.001 for CPR; aOR = 1.76, 95% CI = 1.03-3.01, *P* = 0.039 for LBR). However, no significant difference in EMR was observed between morphological grading groups (18.87% vs. 22.86%, *P* = 0.755). The multivariable models accounted for maternal age, BMI, AMH levels, number of previous miscarriages, endometrial preparation protocols, endometrial thickness, and blastocyst developmental stage/morphological grading.

We further evaluated the joint effects of developmental stage and morphological grading on pregnancy outcomes (**Supplementary Table 3**). Morphological grading was found to exert a significant influence on pregnancy outcomes, particularly among D6 blastocysts.

We stratified the data into two groups based on maternal age (≥35 vs. <35 years) and compared the basic parameters of FET cycles (**Supplementary Table 4**). The results showed that only the number of previous miscarriages differed significantly between the two groups, while no significant differences were observed in maternal BMI, endometrial preparation protocols, endometrial thickness, embryo developmental stage, or morphological grading. Additionally, we analyzed the impact of embryo morphological grading and developmental stage on pregnancy outcomes within each age stratum (**Supplementary Table 5**). For < 35 women, good-quality blastocysts significantly increased CPR versus poor-quality (74.77% vs. 57.78%, *P* = 0.010; aOR = 2.17). For ≥ 35 women, good-quality blastocysts significantly improved LBR (55.56% vs. 28.21%, *P* = 0.018; aOR = 4.56). Embryo developmental stage did not affect EMR or LBR/CPR in either group.

### BCT cycles vs. normal cycles

A comparison of baseline characteristics and ovarian stimulation parameters between BCT and normal cycles revealed significant differences in maternal and paternal age, number of prior pregnancies and miscarriages, COS protocol type, duration of Gn use, blastocyst formation rate, and number of euploid embryos yield per cycle (**Supplementary Table 6**). Comparison of FET cycle characteristics and pregnancy outcomes between BCT and normal cycles further showed significant differences in maternal age, BMI, number of previous miscarriages, and blastocyst morphological grading. No other variables differed significantly (**Supplementary Table 7**).

## Discussion

This study focused on assessing the effects of blastocyst developmental stage (D5 vs. D6) and morphological grading on pregnancy outcomes after SE-FBT in young RPL patients, either carriers of BCT or with normal karyotypes. Among BCT carriers, it was observed that D5 euploid blastocysts significantly outperformed D6 blastocysts in terms of CPR and LBR. However, blastocyst morphology was not found to have a statistically significant effect on reproductive outcomes. Conversely, in karyotypically normal RPL patients, the blastocyst’s developmental stage did not significantly correlate with CPR or LBR. Instead, blastocysts with good morphological grading demonstrated notably better CPR and LBR compared to those with poor morphology. To our knowledge, this represents the first report of such an association.

The association between euploid blastocyst morphological grading and pregnancy outcomes remains controversial in current research. Capalbo et al. (12) demonstrated comparable implantation potential across varying euploid blastocyst morphological grading and developmental stages following PGT-A, a finding corroborated by Viñals Gonzalez et al. (14). Notably, these studies involved AMA populations with mean ages of 37.8 and 38.6 years at oocyte retrieval, respectively. In younger populations, Ji et al. (13) reported no significant impact of euploid blastocyst characteristics on CPR and LBR in PGT-A candidates with a mean maternal age of 30 years. Conversely, Lou et al. (9) observed superior implantation rates in good quality euploid blastocysts (AA/AB/BA; n = 51) compared to poorer quality counterparts (AC/BC; n = 109) among patients under 35 years undergoing PGT-A, though limited by a relatively small sample size. Cimadomo et al. (10) identified significant morphological influences on pregnancy outcomes in a cohort with median maternal age of 39.6 years, where > 90% of PGT-A cases were for AMA. In addition, the high percentage of good quality blastocysts from AMA patients may be questionable. Irani et al. (11) further reported morphological correlations with outcomes, though their study population included all infertility patients receiving routine PGT-A. Our investigation focusing on young RPL patients revealed differential associations based on parental chromosomal status: blastocyst morphological grading correlated with pregnancy outcomes in normokaryotypic cases but not in BCT carriers.

The impact of euploid blastocyst developmental stage on pregnancy outcomes continues to generate debate in the field. Among young patients (< 35 years) undergoing PGT-M/A, Liu et al. (18) reported no significant association between developmental stage and pregnancy outcomes. Conversely, Chen et al. (15) found that developmental stage was associated with implantation rate and LBR, with a similar-aged cohort (mean maternal age 30 years) receiving PGT/A/M/SR. Notably, both studies lack explicit clarification regarding single versus multiple FBT cycles per patient - a critical methodological consideration, as repeated cycles from the same individual may violate statistical independence assumptions. This analytical concern was addressed in Li et al.’s investigation (16), which exclusively analyzed first FBT cycles post-PGT-A and identified significant correlations between developmental stage and LBR. Recent evidence from PGT-M/SR populations (median age 29.7 years) further supports developmental stage as a predictor of LBR (17). The present study found that in the young RPL patients with BCT, blastocyst developmental stage was associated with the pregnancy outcome, whereas in the young RPL patients with normal karyotypes, blastocyst developmental stage had no effect on pregnancy outcome.

Multiple factors may contribute to the discrepancies observed across studies investigating euploid blastocyst transfer outcomes. Foremost, substantial heterogeneity exists in patient populations undergoing PGT. Compared to cohorts undergoing PGT-M/SR, RPL and/or RIF populations present multifactorial etiologies beyond embryonic chromosomal abnormalities. Notably, endometrial receptivity alterations in RIF patients have been well-documented as a key confounding factor (28). Furthermore, the age-related decline in reproductive potential not only correlates with increased aneuploidy rates but also reflects complex metabolic and epigenetic modifications in embryos (21).

Secondly, the inherent subjectivity of embryo grading systems warrants consideration. While intra-laboratory consistency can be achieved through standardized protocols (e.g. Gardner criteria), significant inter-center variability persists in morphological assessments (29). Thirdly, technical variables in trophectoderm biopsy procedures require scrutiny. Operator expertise and ongoing quality control measures are critical, as suboptimal biopsy techniques may compromise embryo viability - evidenced by studies demonstrating reduced implantation potential with excessive trophectoderm cell removal (> 10 cells) (8, 30). Finally, methodological variations in study design merit attention. Additionally, the inclusion of multiple embryo transfer cycles per patient without appropriate statistical adjustment for repeated measures could inflate type I error rates.

This study revealed that in young RPL patients, the impact of euploid blastocyst morphological grading and developmental stage on pregnancy outcomes is contingent upon parental chromosomal status. In cases with karyotypically normal patients, good-quality euploid blastocysts likely possess optimized cellular differentiation mechanisms, mitochondrial homeostasis, and metabolic activity that enhance embryonic implantation potential (31). While D6 blastocysts might reflect transient metabolic fluctuations rather than intrinsic developmental compromise, thereby showing comparable clinical outcomes to D5 blastocysts (32). Conversely, euploid embryos from BCT carriers may still have microdeletions/duplications, and these abnormalities may be reflected by the embryo developmental stage rather than morphological grading. D5 euploid blastocysts may reflect a more complete chromosomal structural repair capacity. On day 5, blastocysts exhibited a substantially higher level of mitochondrial DNA (mtDNA) compared to those on day 6. This suggests that mtDNA quantity might play a crucial role in determining the development rate of blastocysts (33).

Notably, the early miscarriage rate observed in the normal cycles group (20.45%, 36/176) warrants careful consideration. Analysis revealed no association with embryo parameters (developmental stage/morphological grading), endometrial thickness, and endometrial preparation protocols. Crucially, a history of RPL emerged as a strong predictor of miscarriage risk, with rates escalating significantly alongside prior loss burden: 14.75% (2 losses), 22.06% (3 losses), 19.35% (4 losses), and 37.50% (≥ 5 losses; *P* = 0.03 for ≥ 5 vs. 2 losses). This dose-dependent relationship, aligning with Ni et al.’s findings (34), suggests RPL itself may induce endometrial pathophysiological changes, potentially creating a vicious cycle of impaired receptivity. While PGT-A circumvents aneuploidy, our data demonstrate persistent endometrial dysfunction in RPL patients, evidenced by miscarriage rates approaching 37.50% in severe cases – exceeding the 4.05% - 11.02% rates in some PGT-SR studies (35–37). Thus, RPL history is a key indicator of euploid miscarriage risk, underscoring the imperative to address endometrial receptivity in this high-risk population.

Moreover, in the comparison of baseline characteristics between the two groups, the karyotypically normal group reported a higher number of previous miscarriages compared to the BCT group. This difference could also contribute to the distinct impacts of developmental stage and morphological grading of euploid blastocysts on pregnancy outcomes observed between the two groups.

### Strengths and limitations

Numerous studies have examined the impact of blastocyst developmental stage and morphological grading on the LBR after SE-FBT. However, these investigations typically combined heterogeneous populations with various PGT indications (e.g. AMA, RIF, RPL, etc.). In contrast, our study specifically focused on a relatively homogeneous young RPL population and further stratified the analysis based on parental karyotype status (normal karyotype vs. BCT). Additionally, the single-center design ensured consistent embryo scoring throughout the study.

Despite rigorous efforts to minimize bias, this study has several inherent limitations. Its retrospective design carries unavoidable risks of bias, while the single-center setting and limited sample size constrain statistical power and generalizability. Additionally, significant differences in baseline characteristics were observed among cycles with BCT versus normal karyotypes, necessitating consideration of population-level confounding factors as a potential influence on the outcomes. We acknowledge the preliminary nature of these findings; future validation through multicenter studies with larger cohorts is warranted to establish robust evidence-based clinical guidance.

## Conclusion

In young RPL patients with BCT, the developmental stage rather than morphological grading of euploid blastocysts demonstrates significant impact on pregnancy outcomes. In contrast, in karyotypically normal RPL patients, blastocyst morphological grading supersedes developmental stage as the predominant prognostic factor. These findings provide valuable guidance for clinical decision-making regarding embryo selection: 1) For BCT carriers, priority should be given to transferring euploid blastocysts achieving expansion by day 5; 2) For patients with normal karyotypes, morphological assessment should guide selection of optimal quality blastocysts regardless of developmental day. Future foundational studies are imperative to delineate the mechanisms through which parental chromosomal status modulates the association between euploid blastocyst morphological grading and developmental stage with subsequent pregnancy outcomes.

## Data Availability

The raw data supporting the conclusions of this article will be made available by the authors, without undue reservation.
