# Supplementary material for: Impact of euploid blastocyst developmental stage and morphological grading on pregnancy outcomes in young recurrent pregnancy loss patients: association with parental chromosomal status
Source: Front Endocrinol (Lausanne). 2025 Sep 19;16:1644773. doi: 10.3389/fendo.2025.1644773 (PMC12490980; doi:10.3389/fendo.2025.1644773)
Supplement: Supplementary file 2 [file Table2.docx]

**Supplementary Table 2** Logistic regression analysis of blastocyst developmental stage and morphological grading affecting pregnancy outcomes in RPL patients with balanced chromosomal translocations.

|  | Outcome | OR (95% CI) | *P* value | aOR (95% CI) | *P* value |
| --- | --- | --- | --- | --- | --- |
| **CPR**, n (%) |  |  |  |  |  |
| **Good-quality** |  |  |  |  |  |
| D5 | 35/40 (87.50) | 2.52 (0.74-8.61) | 0.140 | 3.52 (0.86-14.41) | 0.0800 |
| D6 | 25/34 (73.53) | ref |  | ref |  |
| **Poor-quality** |  |  |  |  |  |
| D5 | 25/32 (78.13) | 2.57 (1.05-6.29) | 0.039 | 2.34 (0.84-6.54) | 0.105 |
| D6 | 41/71(57.75) | ref |  | ref |  |
| **D5** |  |  |  |  |  |
| Good-quality | 35/40 (87.50) | 1.85 (0.54-6.27) | 0.326 | 1.14 (0.28-4.61) | 0.858 |
| Poor-quality | 25/32 (78.13) | ref |  | ref |  |
| **D6** |  |  |  |  |  |
| Good-quality | 25/34 (73.53) | 2.09 (0.88-4.93) | 0.093 | 2.15 (0.86-5.33) | 0.100 |
| Poor-quality | 41/71 (57.75) | ref |  | ref |  |
| **EMR**, n (%) |  |  |  |  |  |
| **Good-quality** |  |  |  |  |  |
| D5 | 1/35 (2.86) | 0.14 (0.01-1.30) | 0.083 | 0.31 (0.01-13.98) | 0.549 |
| D6 | 4/25 (16.00) | ref |  | ref |  |
| **Poor-quality** |  |  |  |  |  |
| D5 | 4/25 (16.00) | 1.77 (0.40-7.87) | 0.453 | 1.74 (0.38-7.92) | 0.474 |
| D6 | 4/41 (9.76) | ref |  | ref |  |
| **D5** |  |  |  |  |  |
| Good-quality | 1/35 (2.86) | 0.25 (0.01-5.50) | 0.379 | 0.16 (0.02-1.61) | 0.119 |
| Poor-quality | 4/25 (16.00) | ref |  | ref |  |
| **D6** |  |  |  |  |  |
| Good-quality | 4/25 (16.00) | 1.86 (0.43-8.16) | 0.409 | 1.74 (0.37-8.22) | 0.484 |
| Poor-quality | 4/41 (9.76) | ref |  | ref |  |
| **LBR**, n (%) |  |  |  |  |  |
| **Good-quality** |  |  |  |  |  |
| D5 | 33/40 (82.50) | 3.36 (1.19-9.50) | 0.022 | 0.96 (0.27-3.39) | 0.955 |
| D6 | 20/34 (58.82) | ref |  | ref |  |
| **Poor-quality** |  |  |  |  |  |
| D5 | 21/32 (65.63) | 2.12 (0.91-4.96) | 0.082 | 2.77 (1.00-7.63) | 0.049* |
| D6 | 34/71 (47.89) | ref |  | ref |  |
| **D5** |  |  |  |  |  |
| Good-quality | 33/40 (82.50) | 2.43 (0.83-7.06) | 0.104 | 2.19 (0.68-7.00) | 0.187 |
| Poor-quality | 21/32 (65.63) | ref |  | ref |  |
| **D6** |  |  |  |  |  |
| Good-quality | 20/34 (58.82) | 1.56 (0.72-3.41) | 0.260 | 1.70 (0.73-3.94) | 0.217 |
| Poor-quality | 34/71 (47.89) | ref |  | ref |  |

CPR: clinical pregnancy rate; EMR: early miscarriage rate; LBR: live birth rate; OR: odds ratio; CI: confidence interval; aOR: adjusted odds ratio
