# Supplementary material for: Impact of euploid blastocyst developmental stage and morphological grading on pregnancy outcomes in young recurrent pregnancy loss patients: association with parental chromosomal status
Source: Front Endocrinol (Lausanne). 2025 Sep 19;16:1644773. doi: 10.3389/fendo.2025.1644773 (PMC12490980; doi:10.3389/fendo.2025.1644773)
Supplement: Supplementary file 3 [file Table3.docx]

**Supplementary Table 3** Logistic regression analysis of blastocyst developmental stage and morphological grading affecting pregnancy outcomes in normokaryotypic RPL patients.

|  | Outcome | OR (95% CI) | *P* value | aOR (95% CI) | *P* value |
| --- | --- | --- | --- | --- | --- |
| **CPR**, n (%) |  |  |  |  |  |
| **Good-quality** |  |  |  |  |  |
| D5 | 55/74 (74.32) | 1.02 (0.49-2.12) | 0.952 | 0.88 (0.40-1.90) | 0.737 |
| D6 | 51/69 (73.91) | ref |  | ref |  |
| **Poor-quality** |  |  |  |  |  |
| D5 | 30/50 (60.00) | 1.48 (0.70-3.13) | 0.300 | 1.71 (0.79-3.71) | 0.175 |
| D6 | 40/79 (50.63) | ref |  | ref |  |
| **D5** |  |  |  |  |  |
| Good-quality | 55/74 (74.32) | 1.93 (0.85-4.40) | 0.116 | 1.64 (0.72-3.74) | 0.237 |
| Poor-quality | 30/50 (60.00) | ref |  | ref |  |
| **D6** |  |  |  |  |  |
| Good-quality | 51/69 (73.91) | 2.78 (1.43-5.42) | 0.003 | 5.50 (2.23-13.54) | <0.001* |
| Poor-quality | 40/79 (50.63) | ref |  | ref |  |
| **EMR**, n (%) |  |  |  |  |  |
| **Good-quality** |  |  |  |  |  |
| D5 | 10/55 (18.18) | 1.34 (0.36-5.01) | 0.659 | 1.21 (0.33-4.44) | 0.769 |
| D6 | 10/51 (19.61) | ref |  | ref |  |
| **Poor-quality** |  |  |  |  |  |
| D5 | 7/30 (23.33) | 0.99 (0.96-1.02) | 0.656 | 1.07 (0.35-3.23) | 0.911 |
| D6 | 9/40 (22.50) | ref |  | ref |  |
| **D5** |  |  |  |  |  |
| Good-quality | 10/55 (18.18) | 0.73 (0.25-2.17) | 0.571 | 0.85 (0.22-3.28) | 0.81 |
| Poor-quality | 7/30 (23.33) | ref |  | ref |  |
| **D6** |  |  |  |  |  |
| Good-quality | 10/51 (19.61) | 0.08 (0.00-1.46) | 0.088 | 0.95 (0.27-3.31) | 0.930 |
| Poor-quality | 9/40 (22.50) | ref |  | ref |  |
| **LBR**, n (%) |  |  |  |  |  |
| **Good-quality** |  |  |  |  |  |
| D5 | 41/74 (55.41) | 0.83 (0.42-1.64) | 0.599 | 0.76 (0.37-1.55) | 0.452 |
| D6 | 40/69 (57.97) | ref |  | ref |  |
| **Poor-quality** |  |  |  |  |  |
| D5 | 21/50 (42.00) | 1.21 (0.60-2.42) | 0.599 | 1.47 (0.71-3.04) | 0.296 |
| D6 | 31/79 (39.24) | ref |  | ref |  |
| **D5** |  |  |  |  |  |
| Good-quality | 41/74 (55.41) | 1.59 (0.73-3.44) | 0.241 | 2.21 (0.84-5.81) | 0.108 |
| Poor-quality | 21/50 (42.00) | ref |  | ref |  |
| **D6** |  |  |  |  |  |
| Good-quality | 40/69 (57.97) | 2.12 (1.05-4.25) | 0.035 | 2.70 (1.23-5.92) | 0.013* |
| Poor-quality | 31/79 (39.24) | ref |  | ref |  |

CPR: clinical pregnancy rate; EMR: early miscarriage rate; LBR: live birth rate; OR: odds ratio; CI: confidence interval; aOR: adjusted odds ratio
