# Supplementary material for: Impact of euploid blastocyst developmental stage and morphological grading on pregnancy outcomes in young recurrent pregnancy loss patients: association with parental chromosomal status
Source: Front Endocrinol (Lausanne). 2025 Sep 19;16:1644773. doi: 10.3389/fendo.2025.1644773 (PMC12490980; doi:10.3389/fendo.2025.1644773)
Supplement: Supplementary file 4 [file Table4.docx]

**Supplementary Table 4** Characteristics of frozen-thawed transfer cycles in normokaryotypic RPL patients stratified by maternal age.

| Parameters | <35y | ≥35y | *P* value |
| --- | --- | --- | --- |
|  | n=197 | n=75 |  |
| Maternal BMI (kg/m^2^) | 23.14 (20.57,24.91) | 22.41 (21.30,24.69) | 0.944 |
| No. of previous miscarriages (n) |  |  | 0.002* |
| 2 | 67 (34.01) | 21 (28.00) |  |
| 3 | 85 (43.15) | 24 (32.00) |  |
| 4 | 36 (18.27) | 16 (21.33) |  |
| ≥5 | 9 (4.57) | 14 (18.67) |  |
| Endometrial preparation |  |  | 0.76 |
| GnRHa-HRT | 51 (25.89) | 19 (25.33) |  |
| HRT | 135 (68.53) | 50 (66.67) |  |
| NC | 11 (5.58) | 6 (8.00) |  |
| Endometrial thickness (mm) | 8.90 (8.00,10.00) | 8.40 (7.00,9.50) | 0.062 |
| Developmental stage |  |  | 0.826 |
| D5 | 89 (45.18) | 35 (46.67) |  |
| D6 | 108 (54.82) | 40 (53.33) |  |
| Morphological grading |  |  | 0.351 |
| Good quality | 107 (54.31) | 36 (48.00) |  |
| Poor quality | 90 (45.69) | 39 (52.00) |  |
