# Supplementary material for: Impact of euploid blastocyst developmental stage and morphological grading on pregnancy outcomes in young recurrent pregnancy loss patients: association with parental chromosomal status
Source: Front Endocrinol (Lausanne). 2025 Sep 19;16:1644773. doi: 10.3389/fendo.2025.1644773 (PMC12490980; doi:10.3389/fendo.2025.1644773)
Supplement: Supplementary file 5 [file Table5.docx]

**Supplementary Table 5** The association between pregnancy outcomes, blastocyst developmental stage and morphological grading in normokaryotypic RPL patients stratified by maternal age.

|  | Outcome | OR (95% CI) | *P* value | aOR (95% CI) | *P* value |
| --- | --- | --- | --- | --- | --- |
| **<35y n=197** |  |  |  |  |  |
| **CPR**, n (%) |  |  |  |  |  |
| Developmental stage |  |  |  |  |  |
| D5 | 64/89 (71.91) | 1.41 (0.81-2.46) | 0.221 | 1.38 (0.75-2.55) | 0.301 |
| D6 | 68/108 (62.96) | ref |  | ref |  |
| Morphological grading |  |  |  |  |  |
| Good quality | 80/107 (74.77) | 2.16 (1.20-3.87) | 0.010 | 2.17 (1.18-3.99) | 0.013* |
| Poor quality | 52/90 (57.78) | ref |  | ref |  |
| **EMR**, n (%) |  |  |  |  |  |
| Developmental stage |  |  |  |  |  |
| D5 | 12/64 (18.75) | 0.94 (0.35-2.53) | 0.909 | 1.05 (0.26-4.34) | 0.943 |
| D6 | 14/68 (20.59) | ref |  | ref |  |
| Morphological grading |  |  |  |  |  |
| Good quality | 16/80 (20.00) | 1.37 (0.49-3.80) | 0.550 | 1.87 (0.40-8.71) | 0.427 |
| Poor quality | 10/52 (19.23) | ref |  | ref |  |
| **LBR**, n (%) |  |  |  |  |  |
| Developmental stage |  |  |  |  |  |
| D5 | 49/89 (55.06) | 1.27 (0.73-2.19) | 0.395 | 1.31 (0.71-2.42) | 0.395 |
| D6 | 53/108 (49.07) | ref |  | ref |  |
| Morphological grading |  |  |  |  |  |
| Good quality | 61/107 (57.01) | 1.44 (0.77-2.69) | 0.249 | 0.92 (0.43-1.98) | 0.837 |
| Poor quality | 41/90 (45.56) | ref |  | ref |  |
| **≥35y n=75** |  |  |  |  |  |
| **CPR**, n (%) |  |  |  |  |  |
| Developmental stage |  |  |  |  |  |
| D5 | 21/35 (60.00) | 0.97 (0.34-2.78) | 0.954 | 0.85 (0.25-2.82) | 0.787 |
| D6 | 23/40 (57.50) | ref |  | ref |  |
| Morphological grading |  |  |  |  |  |
| Good quality | 26/36 (72.22) | 2.98 (1.00-8.92) | 0.050 | 2.37 (0.75-7.45) | 0.140 |
| Poor quality | 18/39 (46.15) | ref |  | ref |  |
| **EMR**, n (%) |  |  |  |  |  |
| Developmental stage |  |  |  |  |  |
| D5 | 5/21 (23.81) | 3.62 (1.35-9.76) | 0.011 | 1.37 (0.25-7.39) | 0.713 |
| D6 | 5/23 (21.74) | ref |  | ref |  |
| Morphological grading |  |  |  |  |  |
| Good quality | 4/26 (15.38) | 0.29 (0.08-1.01) | 0.051 | 0.51 (0.06-4.72) | 0.555 |
| Poor quality | 6/18 (33.33) | ref |  | ref |  |
| **LBR**, n (%) |  |  |  |  |  |
| Developmental stage |  |  |  |  |  |
| D5 | 13/35 (37.14) | 0.70 (0.27-1.83) | 0.466 | 0.33 (0.09-1.24) | 0.102 |
| D6 | 18/40 (45.00) | ref |  | ref |  |
| Morphological grading |  |  |  |  |  |
| Good quality | 20/36 (55.56) | 3.29 (1.23-8.77) | 0.018 | 4.56 (1.22-17.03) | 0.024* |
| Poor quality | 11/39 (28.21) | ref |  | ref |  |
