# Supplementary material for: Impact of euploid blastocyst developmental stage and morphological grading on pregnancy outcomes in young recurrent pregnancy loss patients: association with parental chromosomal status
Source: Front Endocrinol (Lausanne). 2025 Sep 19;16:1644773. doi: 10.3389/fendo.2025.1644773 (PMC12490980; doi:10.3389/fendo.2025.1644773)
Supplement: Supplementary file 6 [file Table6.docx]

Supplementary Table 6 Comparison of basic clinical characteristics and ovarian stimulation parameters between BCT and normal cycles.

| Parameters | BCT cycles | Normal cycles | *P* value |
| --- | --- | --- | --- |
| Cycles (n) | 149 | 207 |  |
| Maternal age (years) | 29 (26,31) | 32 (30,34) | <0.001* |
| Paternal age (years) | 29 (27,32) | 32 (30,35) | <0.001* |
| Maternal BMI (kg/m^2^) | 23.23 (21.1,25.97) | 22.83 (20.7,24.6) | 0.085 |
| Paternal BMI (kg/m^2^) | 25.46±3.62 | 25.65 (23.2,28.09) | 0.500 |
| AFC (n) | 15 (12,20) | 14 (10,19) | 0.072 |
| Basal FSH (mIU/ml) | 6.39 (5.47,7.27) | 6.29 (5.45,7.1) | 0.686 |
| Basal E_2_ (pg/ml) | 35.17 (28.67,43.96) | 39.18 (30.97,49.28) | 0.149 |
| AMH (ng/ml) | 3.53 (2.52,5.09) | 3.25 (2.02,4.72) | 0.321 |
| No. of prior pregnancies (n) |  |  |  |
| 2 | 82 (55.03) | 40 (19.32) | ref |
| 3 | 39 (26.17) | 76 (36.71) | <0.001* |
| 4 | 16 (10.74) | 40 (19.32) | <0.001* |
| 5 | 6 (4.03) | 23 (11.11) | <0.001* |
| ≥6 | 6 (4.03) | 28 (13.53) | <0.001* |
| No. of previous miscarriages (n) |  |  |  |
| 2 | 97 (65.1) | 70 (33.82) | ref |
| 3 | 36 (24.16) | 83 (40.1) | <0.001* |
| 4 | 11 (7.38) | 36 (17.39) | <0.001* |
| ≥5 | 5 (3.36) | 18 (8.7) | 0.002* |
| PCOS |  |  | 0.126 |
| Yes | 10 (6.71) | 24 (11.59) |  |
| No | 139 (93.29) | 183 (88.41) |  |
| Type of COS protocols |  |  |  |
| Antagonist | 38 (25.5) | 69 (33.33) | 0.034* |
| PPOS | 29 (19.46) | 51 (24.64) | 0.070 |
| Long | 82 (55.03) | 87 (42.03) | ref |
| Total dosage of Gn used (IU) | 2100 (1650,2625) | 2100 (1725,2525) | 0.672 |
| Duration of Gn used (day) | 10 (9,11) | 9 (8,11) | 0.016* |
| E_2_ level on trigger day (pg/ml) ^a^ | 1810 (1171,2615) | 1700 (1112,2450) | 0.530 |
| Endometrial thickness on trigger day (mm) | 9 (7,10) | 9 (7,10) | 0.226 |
| No. of oocytes retrieved per cycle (n) | 14 (10,18) | 12 (7,17) | 0.093 |
| MII rate per cycle (%) | 85.71 (73.33,95.24) | 83.33 (71.43,96.55) | 0.876 |
| 2PN rate per cycle (%) | 83.33 (75,93.75) | 83.33 (71.43,100) | 0.332 |
| Cleavages rate per cycle (%) | 90.91 (83.33,100) | 94.44 (80,100) | 0.818 |
| Blastocyst formation rate per cycle (%) | 60 (45.45,75) | 64.29 (50,80) | 0.046* |
| No. of blastocyst biopsied per cycle (n) | 5 (3,7) | 4 (3,6) | 0.130 |
| No. of euploid embryos per cycle (n) | 2 (1,2) | 2 (1,3) | <0.001* |

BMI: body mass index; AFC: antral follicle count; FSH: follicle stimulating hormone; E_2_: estradiol; AMH: anti-Mullerian hormone; PCOS: polycystic ovary syndrome; COS: controlled ovarian stimulation; PPOS: progestin-primed ovarian stimulation; Gn: gonadotropin; PN: pronucleus.

a: A total of 19 BCT cycles and 24 normal cycles with E_2_ levels above 3000 pg/ml and no specific values were recorded and therefore excluded from the statistical description.
