# Supplementary material for: Impact of euploid blastocyst developmental stage and morphological grading on pregnancy outcomes in young recurrent pregnancy loss patients: association with parental chromosomal status
Source: Front Endocrinol (Lausanne). 2025 Sep 19;16:1644773. doi: 10.3389/fendo.2025.1644773 (PMC12490980; doi:10.3389/fendo.2025.1644773)
Supplement: Supplementary file 7 [file Table7.docx]

Supplementary Table 7 Comparison of frozen-thawed embryo transfer cycle characteristics and pregnancy outcomes between BCT and normal cycles.

|  | BCT cycles | Normal cycles | *P* value |
| --- | --- | --- | --- |
| Cycles (n) | 177 | 272 |  |
| Maternal age (years) | 30.00 (27.00,32.00) | 32.00 (30.00,35.00) | <0.001* |
| Maternal BMI (kg/m^2^) | 23.40 (21.45,26.04) | 22.96 (21.01,24.91) | 0.016* |
| No. of previous miscarriages (n) |  |  |  |
| 2 | 115 (64.97) | 88 (32.35) | ref |
| 3 | 43 (24.29) | 109 (40.07) | <0.001* |
| 4 | 12 (6.78) | 52 (19.12) | <0.001* |
| ≥5 | 7 (3.95) | 23 (8.46) | 0.003* |
| Endometrial thickness (mm) | 9.00 (8.00,9.90) | 8.70 (7.90,10.00) | 0.583 |
| Endometrial preparation |  |  |  |
| GnRHa-HRT | 40 (22.60) | 70 (25.74) | 0.280 |
| HRT | 131 (74.01) | 185 (68.01) | 0.227 |
| NC | 6 (3.39) | 17 (6.25) | ref |
| Developmental stage |  |  |  |
| D5 | 72 (40.68) | 124 (45.59) | 0.640 |
| D6 | 105 (59.32) | 148 (54.41) | ref |
| Morphological grading |  |  |  |
| Good quality | 74 (41.81) | 143 (52.57) | 0.025* |
| Poor quality | 103 (58.19) | 129 (47.43) | ref |
| Expansion degree |  |  |  |
| 3 | 8 (4.52) | 1 (0.37) | 0.046* |
| 4 | 139 (78.53) | 220 (80.88) | ref |
| 5 | 15 (8.47) | 35 (12.87) | 0.297 |
| 6 | 15 (8.47) | 16 (5.88) | 0.259 |
| CPR | 126 (71.19) | 176 (64.71) | 0.407 |
| EMR | 13 (10.32) | 36 (20.45) | 0.064 |
| LBR | 108 (61.02) | 133 (48.90) | 0.116 |

GnRHa: gonadotropin-releasing hormone agonist; HRT: hormone replacement treatment; NC: natural cycle; CPR: clinical pregnancy rate; EMR: early miscarriage rate; LBR: live birth rate.
